# Mapping microbiome-redox spectrum and evaluating Microbial-Redox Index in chronic gastritis

**DOI:** 10.1038/s41598-022-12431-x

**Published:** 2022-05-19

**Authors:** Manas Kumar Panigrahi, Venkatesh Kaliaperumal, Abhishek Akella, Giriprasad Venugopal, Balamurugan Ramadass

**Affiliations:** 1 Department of Gastroenterology, All India Institute of Medical Sciences, Bhubaneswar, India; 2 MYAS-NIN Department of Sports Science, ICMR-National Institute of Nutrition, Hyderabad, India; 3 Center of Excellence for Clinical Microbiome Research, All India Institute of Medical Sciences, Bhubaneswar, 751019 India; 4 Department of Biochemistry, All India Institute of Medical Sciences, Bhubaneswar, India

**Keywords:** Biochemistry, Microbiology, Gastroenterology, Risk factors

## Abstract

Peptic ulcer disease (PUD) and chronic gastritis are prevalent in developing countries. The role of oxidative stress in the pathogenesis of gastrointestinal mucosal disorders is well recognized. In PUD, the gastric mucosa and its associated microbiome are subject to diet and stress-induced oxidative perturbations. Tissue redox potential (ORP) measurement can quantify oxidative stress, reflecting the balance between prooxidants and antioxidants. This study hypothesizes that the oxidative stress quantified by tissue ORP will be associated with characteristic changes in the mucosa-associated microbiome in PUD and gastritis. In addition, we propose using relative microbial abundance as a quantitative marker of mucosal health. Endoscopy was performed to obtain gastric mucosal biopsies from ten PUD and ten non-ulcer dyspepsia (NUD) patients. The tissue ORP was measured directly with a microelectrode using a biopsy specimen. A second specimen from an adjacent site was subjected to 16s rRNA gene sequencing. From the OTUs, the relative abundance of the microbial taxon in each of the samples was derived. We analyzed the genome of the predominant species for genes encoding the utilization of oxygen as an electron acceptor in respiration and for the presence of antioxidant defense mechanisms. The organisms were then grouped based on their established and inferred redox traits. Shannon diversity index and Species richness were calculated on rarefied data. The relative abundance of organisms that prefer high ORP over those that favor low ORP is conceived as the “Microbial Redox Index (MRI),” an indicator of mucosal health. In the gastric mucosa, aerobic species predominate and are more diverse than the anaerobes. The predominant aerobes are *Helicobacter pylori* and *Sphingobacterium mizutaii*. The abundance of these two species had an inverse correlation with the abundance of low ORP preferring anaerobes. Their relative abundance ratio (Microbial Redox Index) correlated with the tissue oxidation–reduction potential (ORP), a direct measure of oxidative stress. Correlation analysis also revealed that the abundance of all anaerobes inversely correlated with the dominant aerobic taxa. In addition, Shannon and Species richness diversity indices, the probable indicators of mucosal health, were negatively correlated with Microbial Redox Index. Using PUD as a prototype mucosal disease, this article describes a generalized approach to infer and quantify mucosal oxidative stress by analyzing the relative abundance of microorganisms that preferentially grow at the extremes of the tissue redox potential. This ratiometric Microbial Redox Index can also be assessed using simple qPCR without the need for sequencing. The approach described herein may be helpful as a widely applicable quantitative measure of mucosal health with prognostic and therapeutic implications.

## Introduction

The human gastric mucosa harbors hundreds of different bacterial species^1^. Although *Helicobacter pylori* is implicated in peptic ulcer disease, only 1–10% of individuals carrying *H. pylori* develop the condition^2^. Many endogenous and exogenous factors are involved in gastritis and peptic ulcer disease. This fact suggests the potential role of other microbial flora and physiochemical factors in disease pathogenesis. Oxidation–reduction potential (ORP) or Redox potential (Eh) is a fundamental physiochemical variable like pH. Many redox couples determine the level of the tissue redox potential (e.g., GSH/GSSG, Vit COx/Vit CRed, etc.)^3^. In addition, the intestine has a radial and an axial oxygen gradient which influences the local redox potential and intestinal health along with diet, luminal contents, and the microbiome^4,5^.

Oxidative stress implies an increase in tissue ORP. Irrespective of the etiology of PUD, induction of mucosal oxidative stress is considered to be the primary mechanism by which these factors contribute to the disease progression^6^. Reactive oxygen species (ROS) are classical prooxidants generated by mucosal flora, ingested food, or stress. The host inflammatory response that follows the insult sets off a vicious cycle leading to further oxidative damage. ORP is a direct quantitative measure of the balance between prooxidants and antioxidants^7,8^. The value of the redox potential measured in millivolts depends on the oxidizing or reducing nature of the environment. It is one of the abiotic factors that determine the microbiome composition and diversity, similar to pH^9^. While pH is a well-established master regulator in physiology; the redox potential has not received the attention that it duly deserves due to limitations associated with measuring its value accurately. Therefore, many surrogate markers of redox potential/oxidative stress based on levels of discrete prooxidants or antioxidants are used^10^.

The stomach is a highly oxidizing compartment and is less densely populated^11,12^. There is a gradual increase in microbial density along the intestine. While the gastric fundus has a redox potential of around + 400 to + 500 mV, the colon is anaerobic and has an ORP of − 200 to − 300 mV. There is spatial heterogeneity in the redox potential and microbial composition/density both along and across the lumen of the GI tract^4,13^. Aerobes are closely associated with the mucosal surface in the intestine. Facultative anaerobes and anaerobes are distributed towards the lumen. A similar distribution is likely to be present in the gastric mucosa. However, the relative proportion of the aerobe to anaerobe is much higher here due to the highly oxidative nature of this compartment. Oxidative stress is likely to increase this ratio further. The gastric mucosal aerobe anaerobe ratio measurement may serve as an excellent surrogate estimate of mucosal oxidative stress and mucosal health. This study is based on the hypothesis that oxidative stress associated with PUD will increase the mucosal aerobe to anaerobe ratio. To test our hypothesis, the gastric tissue redox potential and gastric mucosal microbial abundance were analyzed in peptic ulcer and non-ulcer dyspepsia patients.

## Methods

### Study design and participants

This observational cross-section study was carried out in the departments of Biochemistry and Gastroenterology at a teaching and tertiary care hospital in Bhubaneswar, India, during the period 2018–2019. The study protocol was approved by the Institutional review & ethical committee at the All India Institute of Medical Sciences, Bhubaneswar and all procedures involved in this study have been performed in accordance with the Declaration of Helsinki.

This proof-of-concept study included twenty patients diagnosed with dyspepsia or upper gastrointestinal bleed presenting to the gastroenterology department. Informed consent was taken from all the participants for this study. In addition, demographic information was obtained from all patients at the time of recruitment.

### Study procedures

Study participants underwent an upper GI endoscopy to evaluate ulcer status. Upper GI endoscopy revealed ulcerative lesions in 10 patients, while the remaining ten subjects were categorized as non-ulcer dyspepsia. Two biopsies were collected from each patient. If ulcer was present, the biopsies were obtained from mucosa adjacent to the ulcer site.

### Measurement of redox potential

One of the biopsy specimens was used to measure redox potential immediately using a microprobe redox electrode attached to a redox meter (PHORP-XS-Lazer Research Lab). The probe surface was wholly pressed against the tissue during measurement. The other biopsy tissue was snap-frozen in liquid nitrogen and transported in liquid nitrogen for storage at − 80 °C.

### DNA isolation

Biopsy specimens were collected using 2 ml sterile microfuge tubes in liquid nitrogen and immediately stored in a − 80 °C freezer. DNA from tissue was extracted using QIAamp DNA Blood Mini Kit following the manufacturer’s guidelines. The DNA was eluted using 200 µl using nuclease-free water and stored in a − 80**°** freezer until amplification and sequencing. DNA was quantitated using Nanodrop and Qubit fluorimeter for quality check.

### 16s rRNA gene sequencing and analysis

Using 25 ng of extracted DNA, the bacterial 16S rRNA hypervariable region V3–V4 was amplified. The reaction included KAPA HiFi HotStart Ready Mix and 100 nm final concentration of modified 341F (V3V4F 5′ CCTACGGGNGGCWGCAG 3′) 785R (V3V4R 5′ GACTACHVGGGTATCTAATCC 3′) primers. The PCR involved an initial denaturation of 95 °C for 5 min followed by 25 cycles of 95 °C for 30 s, 55 °C for 45 s, and 72 °C for 30 s, and a final extension at 72 °C for 7 min. The amplicons were purified using Ampure beads to remove unused primers. Additional 8 cycles were performed using Illumina barcoded adapters to prepare the sequencing libraries.

The sequence data quality was checked using FastQC and MultiQC software. The data was checked for base call quality distribution, % bases above Q20, Q30, %GC, and sequencing adapter contamination. All the samples passed the QC threshold (Q20 > 95%). The reads were trimmed (20 bp) from the 5′ end to remove the degenerate primers. The trimmed reads were processed to remove adapter sequences and low-quality bases using Trimgalore. The QC passed reads were imported into Mothur, and the pairs were aligned to form contigs. The contigs were screened for errors, and only those between 300 and 532 bp were retained. Any contig with ambiguous base calls was rejected. The high-quality contigs were checked for identical sequences, and duplicates were merged. The filtered contigs were processed and classified into taxonomical outlines based on the GREENGENES v.13.8-99 database. The contigs were then clustered into OTUs (Operational Taxonomic Unit). After the annotation, OTU abundance was estimated for each species and genera. Alpha diversity was evaluated using observed, Chao1, Fischer, Simpson, and Shannon indexes, and then a statistical ANOVA was used to detect the index value between the two groups. Beta diversity at the species level was evaluated using the PCoA method with the Jensen–Shannon divergence distance method. Alpha diversity estimators and beta-diversity metrics were computed in an online microbiome data analysis platform (Microbiome Analyst) (https://www.microbiomeanalyst.ca/MicrobiomeAnalyst)^14^.

The top 35 species accounting for 88.5% of the total counts were natural log-transformed and sample normalized. The relative abundance of each species in all the samples was obtained. The bivariate Spearman correlation matrix was computed using SPSS on the relative abundance matrix. The correlation matrix was color-coded based on its value. We performed a bivariate Pearson correlation analysis on the species abundance matrix to infer the association between tissue redox potential, pH of gastric juice, and ulcer/bleeding on specific organisms.

### Redox categorization of species based on oxygen utilization

From published literature, the phenotype of the organism for oxygen requirement/tolerance of the predominant 35 species were deduced, and organisms were categorized as aerobes or anaerobes (Table 2). Obligate aerobes, microaerophiles, and facultative anaerobes were considered aerobes in our broad categorization. The genome sequence of the species was analyzed for genes encoding oxygen utilization and anti-oxidant defense. The species' genome sequence information and features were obtained from Bacterial Diversity Metadatabase BacDive^15^ through Genbank or PATRIC database. The presence of oxygen utilizing genes for aerobic cytochrome oxidase and microaerobic cytochrome oxidase as described by Ravcheev et al.^16^, was noted. The presence of the following anti-oxidant defense genes against superoxide and hydrogen peroxide, namely superoxide dismutase, catalase, and superoxide reductase/rubredoxin, was documented. Based on phenotypic and genotypic features, the organisms were categorized into five redox groups (Table 2). Further details regarding the classification are discussed in the results section.

### Calculation of Shannon diversity index (H) and species richness

The 729 species identified in the twenty samples were rarefied using Paleontological Statistics Package PAST 3.25^17^. Shannon diversity, and species richness were calculated for a rarified sample size of 1701.

### Ethics approval and consent to participate

Institutional Ethics Committee clearance (IM-F/06/2017) from AIIMS, Bhubaneswar, India.

### Consent for publication

All authors of this work concur with this submission, and the data presented have not been previously reported, nor are they under consideration for publication elsewhere.

## Results

### Patient characteristics

The patients who presented with dyspepsia or GI bleeding were classified into two groups of ten subjects, each based on the presence or absence of an ulcer on endoscopy (Table 1). The mean age of the ulcer group was 44.3 ± 12.33 (mean ± SD), and the non-ulcer group was 40.36 ± 9.70 (mean ± SD). Two enrolled patients had presented with Upper GI bleeding.

**Table 1:** Demographic and clinical characteristics of the study subjects.

Characteristics	Ulcer group (n = 10)	Non-ulcer group (n = 10)
Age (years ± SD)	44.3 ± 12.33	40.36 ± 9.70
Gender (male/female)	9/1	7/3
**Clinical presentation**:		
Dyspepsia	8/10	10/10
Upper G.I Bleeding	2/10	0/10
**Upper G I endoscopy**:		
Duodenal ulcer	8/10	0
Pyloric channel ulcer	2/10	0
Antral erosions	0	5/10
Corporal and antral erythema	0	3/10
Fundal and corporal erythema	0	2/10

### Redox spectrum of the gastric mucosal microbiome

The 16s rRNA V3–V4 region amplification and sequencing of the gastric biopsy tissue DNA yielded 2054 OTUs. These were annotated to 729 species. For relative abundance analysis, the top 35 species were used. The aerobic organisms consisting of obligate aerobes, microaerophilic and facultative anaerobes outnumbered the anaerobes. The most common species observed were *Propionibacterium acnes, Enterococcus hemoperoxidus,* and *Helicobacter pylor*i. Aerobic organisms requiring high oxygen levels (high redox potential) possess low-affinity aerobic cytochrome oxidases (Cco, Cta, Qox, Cyo)^16^. Organisms that possess high-affinity microaerobic cytochrome oxidases (Cyd, Cyf) express them in an environment having low oxygen^16,18^. Aerobes were categorized into two groups based on the redox properties (Table 2). Group I consists of organisms with low-affinity aerobic cytochrome oxidases (e.g., *Helicobacter pylori, Sphingobacterium mizutaii, Thermomonas fusca,* etc.). Hence their abundance reflects highly oxygenated tissue with higher redox potential. Group II aerobes express only microaerobic cytochrome oxidases (*Enterococcus hemoperoxidus, Streptococcus agalactiae*, and *Rothia mucilaginosa*). These organisms can thrive only under low oxygen tension. An abundant anaerobic species, *Propionibacterium acnes,* is unique in having both high and low-affinity cytochrome oxidases (Group III). The anaerobes having genes coding only for microaerobic cytochromes are classified under group-IV. *Akkermansia muciniphila, Bacteroides acidifaciens, Parabacteroides distasonis, Lactococcus garvieae, and Prevotella copri species* are group-IV anaerobes having high-affinity microaerobic cytochrome oxidases*.* They do not have genes coding for low-affinity cytochrome oxidase. Anaerobes belonging to group V-a (*Clostridium intestinale*, *Clostridium perfringens,* and *Butyricicoccus pullicaecorum*) are devoid of both high and low-affinity cytochrome oxidases but have genes encoding non-heme catalase. The non-heme catalase helps growth and survival in an environment with high redox potential^19^. The group V-b anaerobes (*Faecalibacterium prausnitzii* and *Bifidobacterium longum*) have neither microaerobic cytochrome oxidases nor catalase. These two species can be considered to lie at the lower end of the tissue ORP, and hence their abundance indicates a highly reducing environment. The relative abundance of the top two predominant aerobic Group-I species over the other anaerobic species in groups III, IV, and V is calculated as the Microbial Redox Index (MRI). This ratio for each sample was regressed and correlated with the tissue redox potential, Shannon diversity index, and Species richness.

**Table 2:** Redox categorization of gastric mucosal bacteria.

S. no.	OTU abundance ranking	Organism	Redox group	Phenotype	Aerobic cytochrome oxidase	Microaerobic cytochrome	SOD	Catalase	SOR/rubredoxin
1	3	*Helicobacter pylori*	I	Aerobe	+	0	+	+	0
2	6	*Sphingobacterium mizutaii*	I	Aerobe	+	+	+	+	+
3	7	*Thermomonas fusca*	I	Aerobe	+	0	+	+	+
4	9	*Acinetobacter guillouiae*	I	Aerobe	+	+	+	+	+
5	10	*Brevundimonas vesicularis*	I	Aerobe	+	+	+	+	0
6	12	*Enhydrobacter aerosaccus*	I	Aerobe	+	+	+	+	0
7	14	*Acinetobacter lwoffii*	I	Aerobe	+	+	+	+	+
8	16	*Paenibacillus amylolyticus*	I	Aerobe	+	+	+	+	0
9	17	*Pseudoalteromonas luteoviolacea*	I	Aerobe	+	+	+	+	0
10	18	*Vibrio harveyi*	I	Aerobe	+	+	+	+	0
11	19	*Sulfuricurvum kujiense*	I	Aerobe	+	+	+	0	0
12	21	*Bacillus marisflavi*	I	Aerobe	+	+	+	+	0
13	22	*Paracoccus marcusii*	I	Aerobe	+	+	+	+	0
14	24	*Thalassospira xiamenensis*	I	Aerobe	+	+	+	+	0
15	25	*Paracoccus aminovorans*	I	Aerobe	+	+	+	+	0
16	26	*Pseudomonas caeni*	I	Aerobe	+	+	+	+	0
17	31	*Burkholderia gladioli*	I	Aerobe	+	+	+	+	+
18	2	*Enterococcus hemoperoxidus*	II	Aerobe	0	+	+	+	0
19	4	*Rothia mucilaginosa*	II	Aerobe	0	+	+	0	0
20	5	*Streptococcus agalactiae*	II	Aerobe	0	+	+	0	0
21	1	*Propionibacterium acnes*	III	Anaerobe	+	+	+	+	0
22	8	*Akkermansia muciniphila*	IV	Anaerobe	0	+	+	+	0
23	13	*Prevotella copri*	IV	Anaerobe	0	+	0	0	+
24	27	*Lactococcus garvieae*	IV	Anaerobe	0	+	+	0	0
25	30	*Bacteroides acidifaciens*	IV	Anaerobe	0	+	+	+	+
26	33	*Prevotella melaninogenica*	IV	Anaerobe	0	+	0	0	+
27	35	*Parabacteroides distasonis*	IV	Anaerobe	0	+	+	+	0
28	20	*Clostridium perfringens*	V-a	Anaerobe	0	0	+	+	+
29	23	*Clostridium intestinale*	V-a	Anaerobe	0	0	+	+	+
30	29	*Butyricicoccus pullicaecorum*	V-a	Anaerobe	0	0	0	+	+
31	28	*Faecalibacterium prausnitzii*	V-b	Anaerobe	0	0	0	0	0
32	34	*Bifidobacterium longum*	V-b	Anaerobe	0	0	0	0	0

### Correlation between tissue ORP, Shannon index, Species richness, and Microbial Redox Index

**Figure 1:**
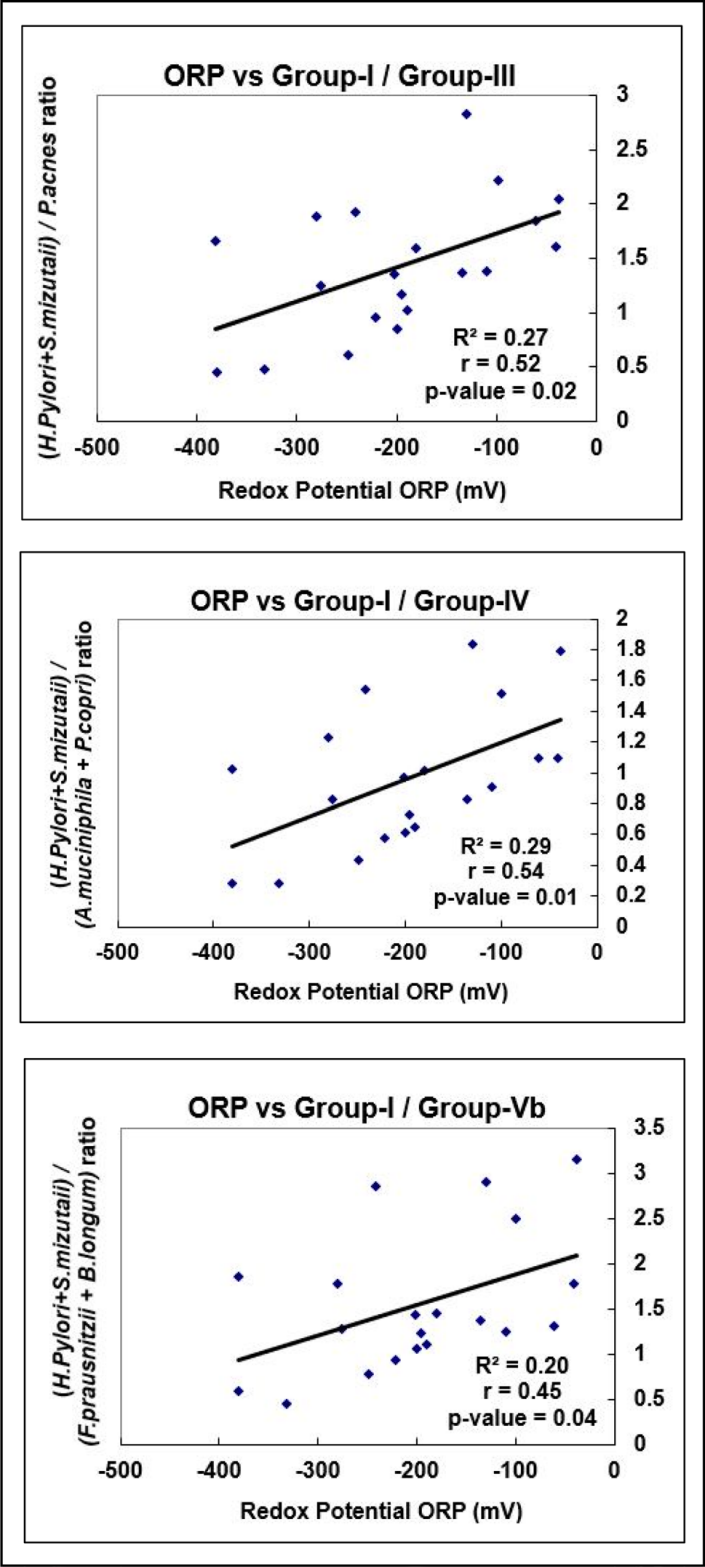
Linear regression analysis of Microbial Redox Index with tissue ORP.

**Figure 2:**
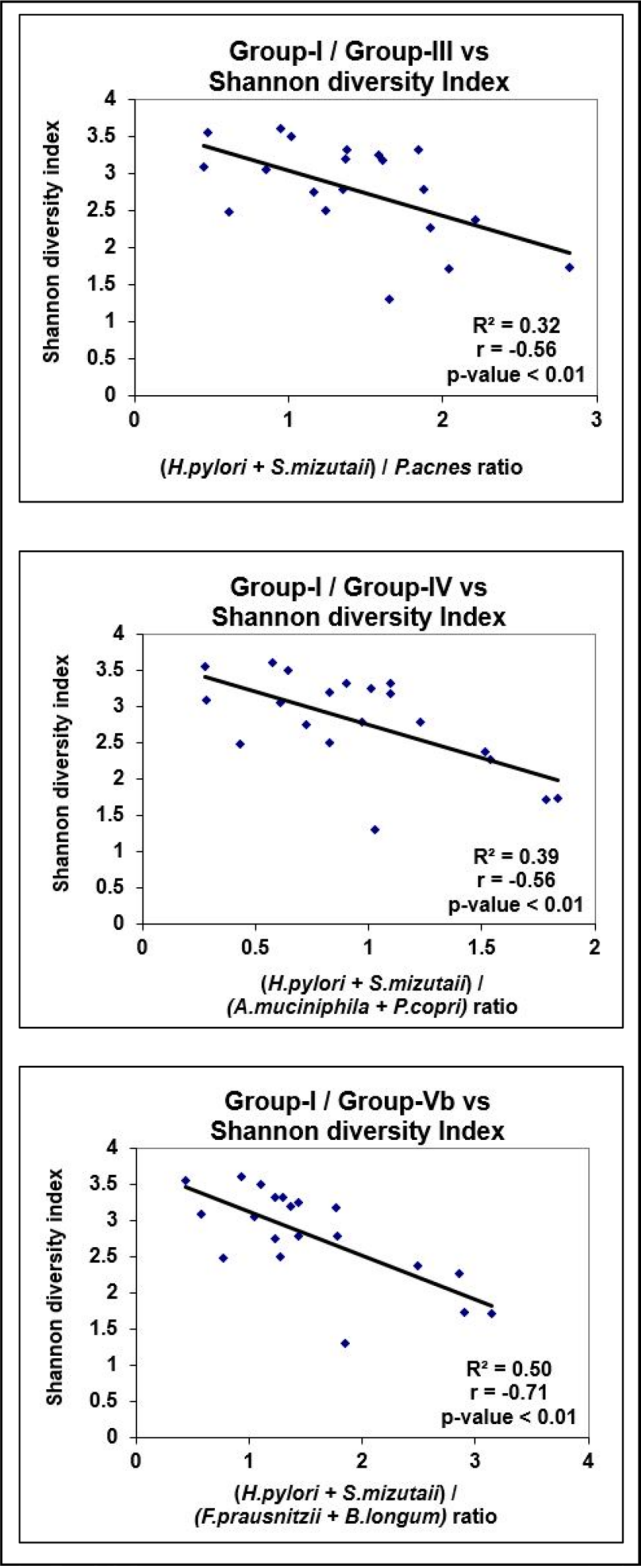
Linear regression analysis of Microbial Redox Index with Shannon Index.

**Figure 3:**
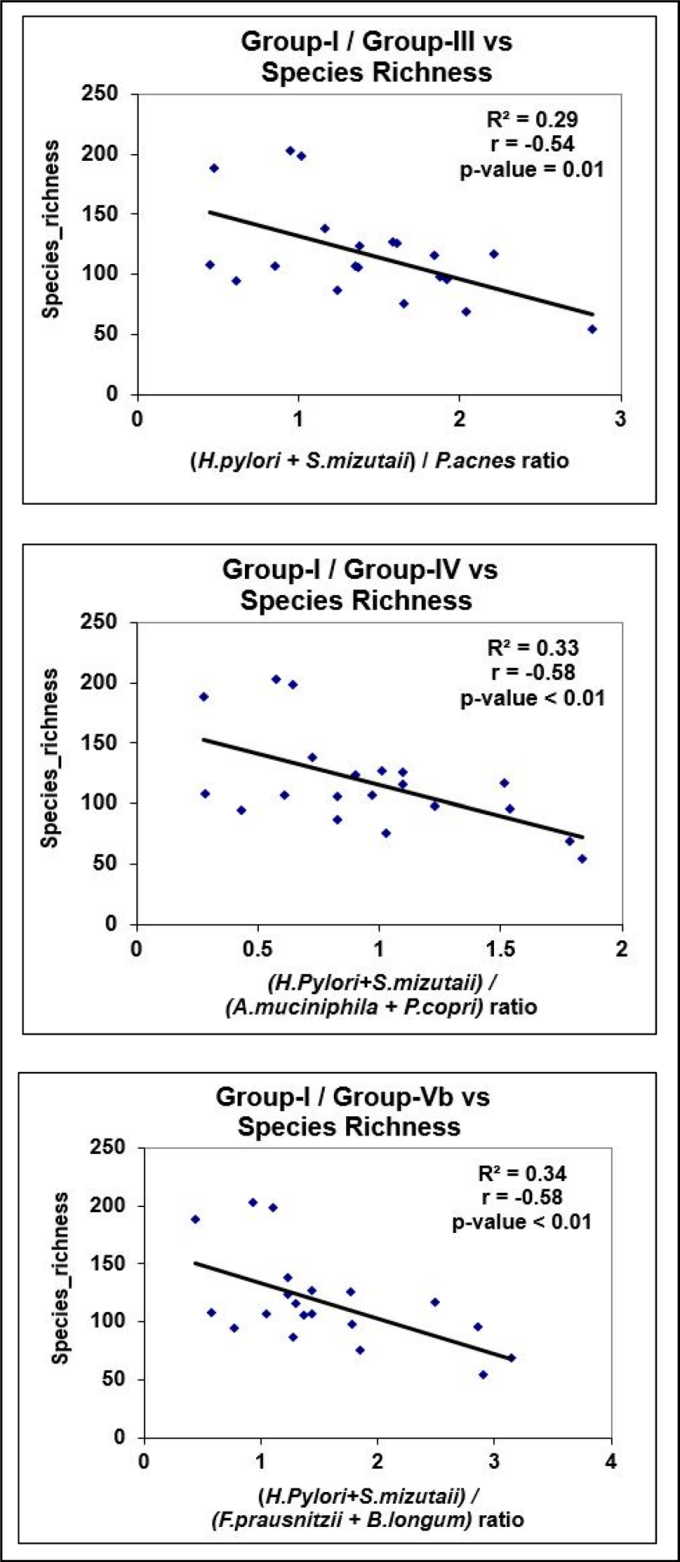
Linear regression analysis of Microbial Redox Index with Species richness.

Linear regression analysis of the measured tissue redox potential showed a significant positive correlation with the Microbial Redox Index (Fig. 1). This index is obtained by calculating the ratio of the two highest abundance Group-I aerobic species (*H. pylori* and *S. mizutaii*) over the anaerobic species in Group-III (*P. acnes*), Group-IV (*A. muciniphila and P. copri*) and Group-Vb separately (Fig. 1). Conversely, a negative correlation was observed between the Shannon index, Species richness, and the Microbial Redox Index (Figs. 2, 3).

### Redox mapping of the gastric mucosal microbiome

**Figure 4:**
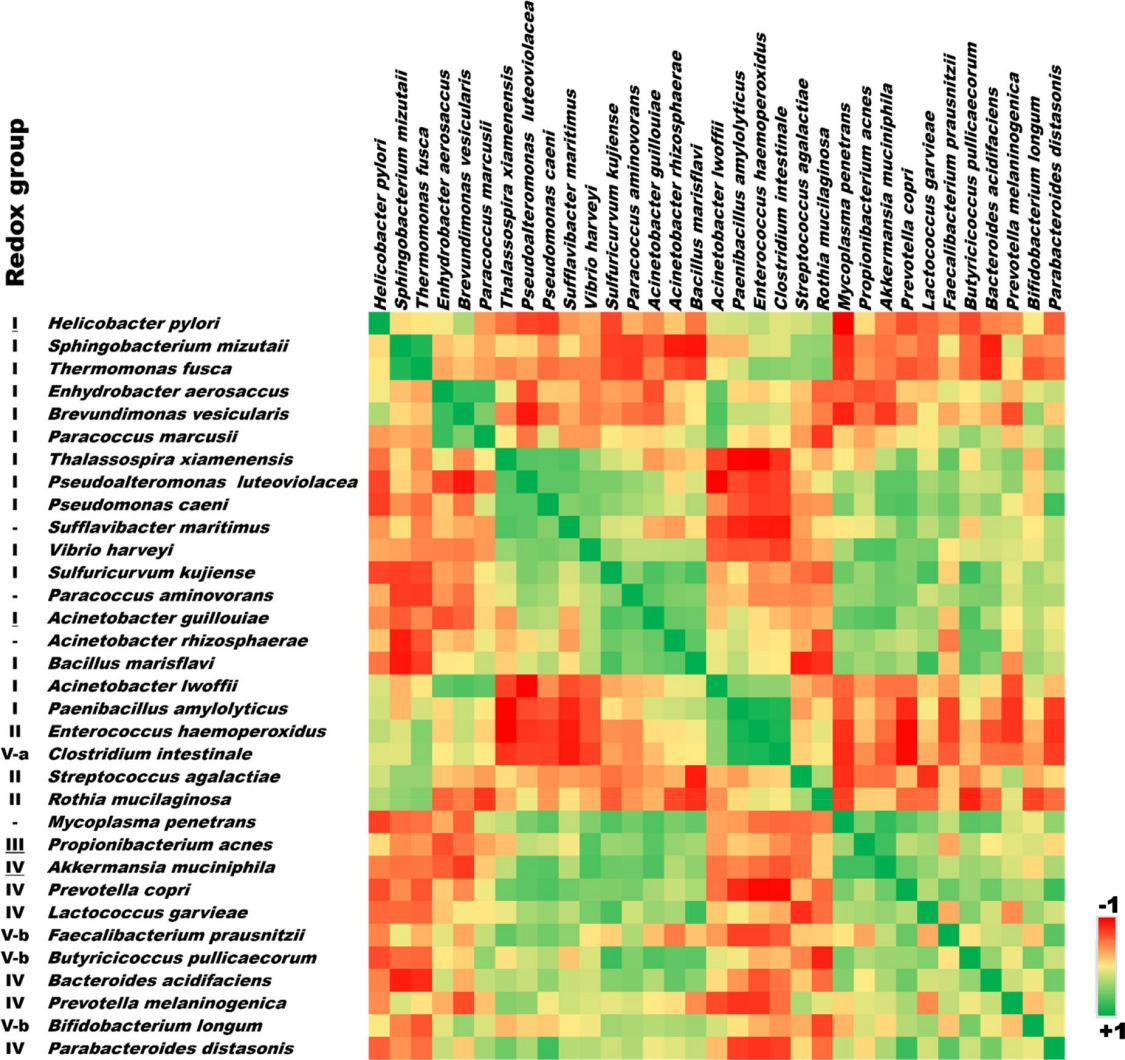
Color-coded Spearman correlation matrix of gastric mucosa-associated species.

**Figure 5:**
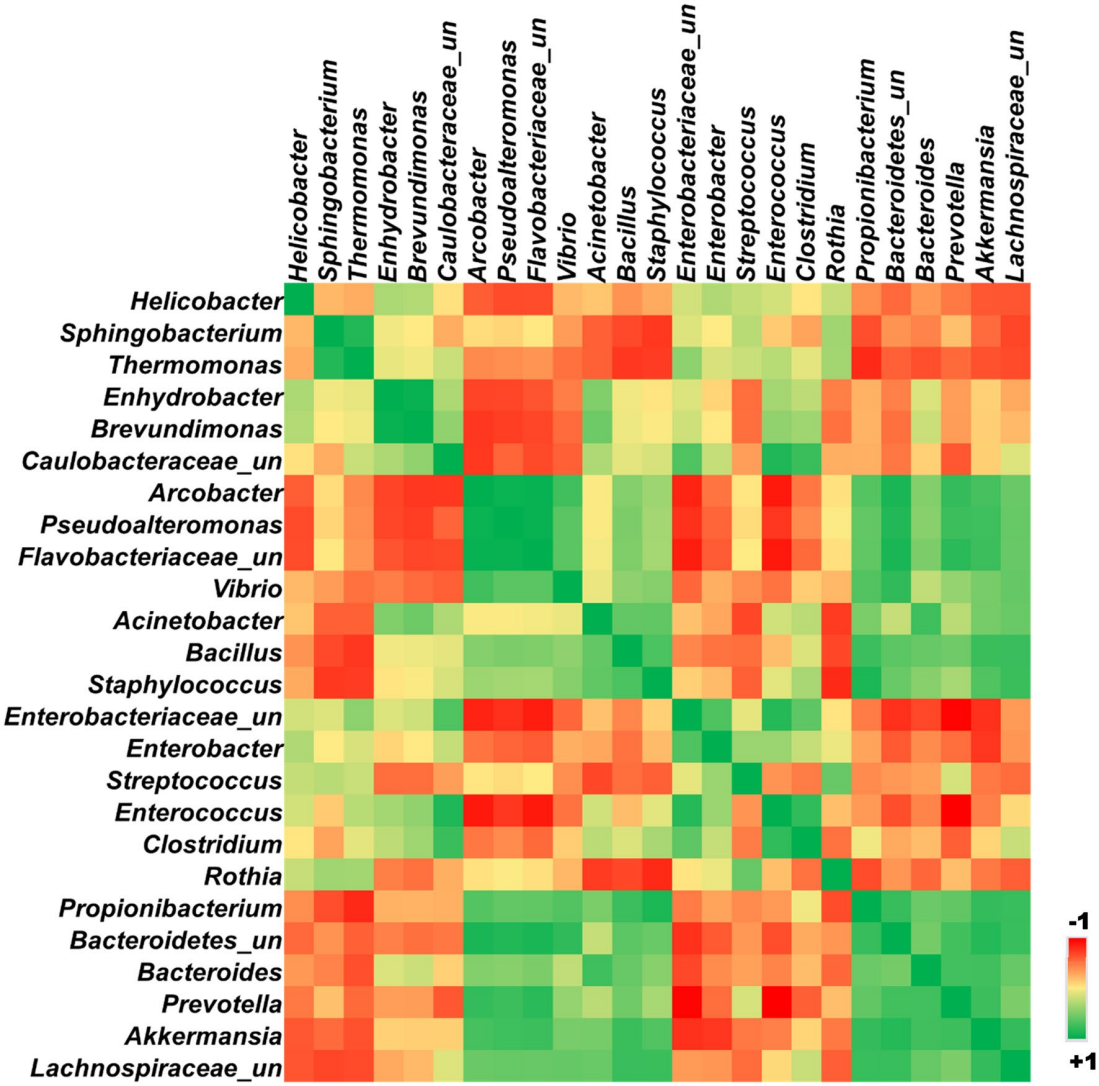
Color-coded Spearman correlation matrix of the relative abundance of gastric mucosa-associated genera.

The color-coded Spearman correlation matrix of the predominant species showed some interesting patterns with respect to the redox groups (Fig. 4). The group-III, IV, and V anaerobes inversely correlated with the most abundant Group-I aerobes and Group II aerobes. There is a positive correlation between dominant Group-I aerobes and Group-II aerobes. The low abundance Group-I aerobes showed a positive correlation with anaerobes. The distribution of aerotolerant *Clostridium intestinale* possessing SOD and catalase activity was more closely correlated to Group-II aerobes than with anaerobes. The redox group-based inferences made at the species level reflected similar correlation analysis and mapping performed on the genera (Fig. 5).

### Microbial species correlations with ulcer, tissue ORP, gastric juice pH, and GI bleed

**Figure 6:**
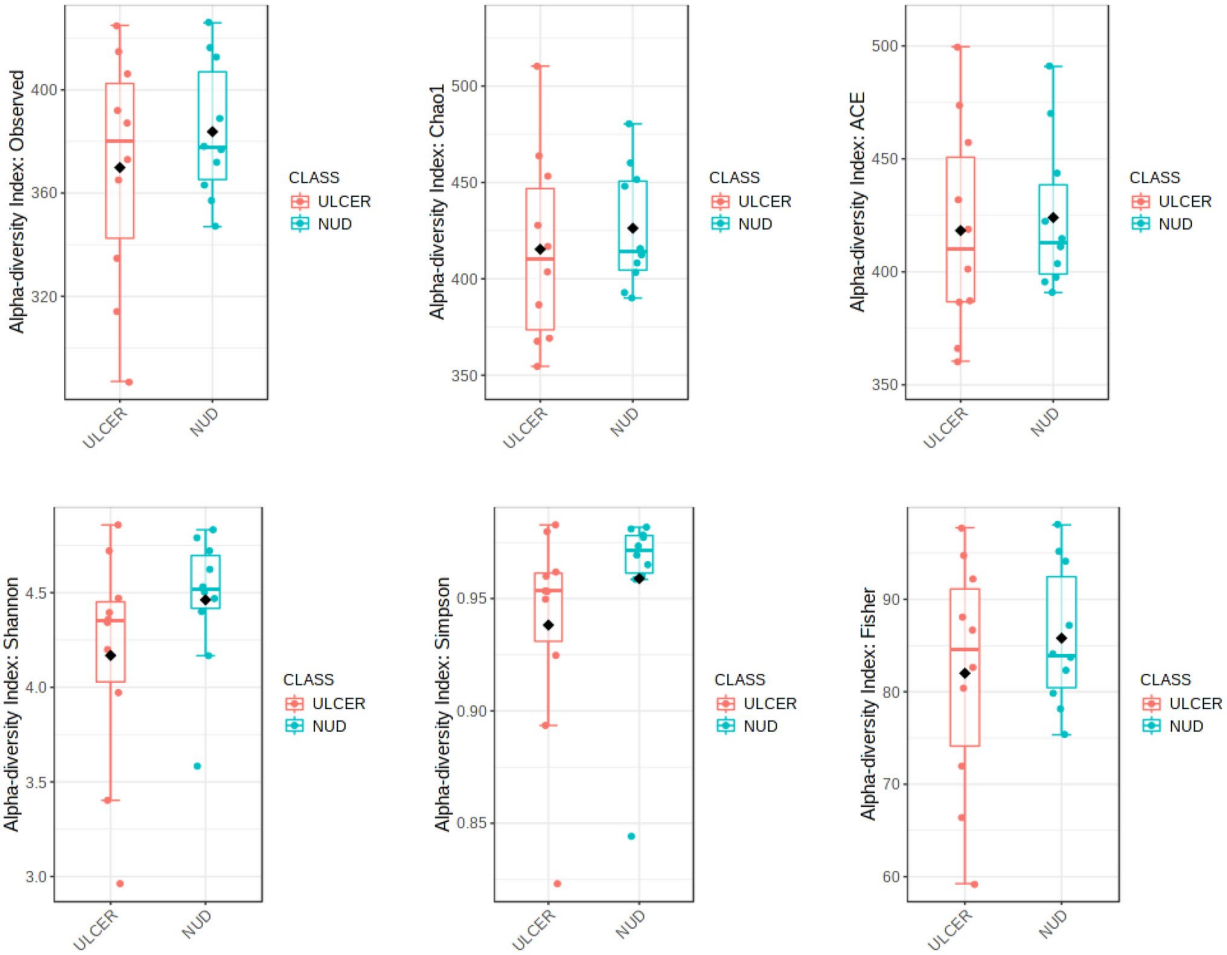
Comparison of alpha diversity indices in ulcer and non-ulcer microbiome at Species-level represented as a boxplot. Each boxplot represents diversity distribution within the study group, and are statistically not significant [Observed (p = 0.87), Chao1 (p = 0.42), ACE (p = 0.53), Shannon (p = 0.94), Simpson (p = 0.84), and Fischer (p = 0.87)].

**Figure 7:**
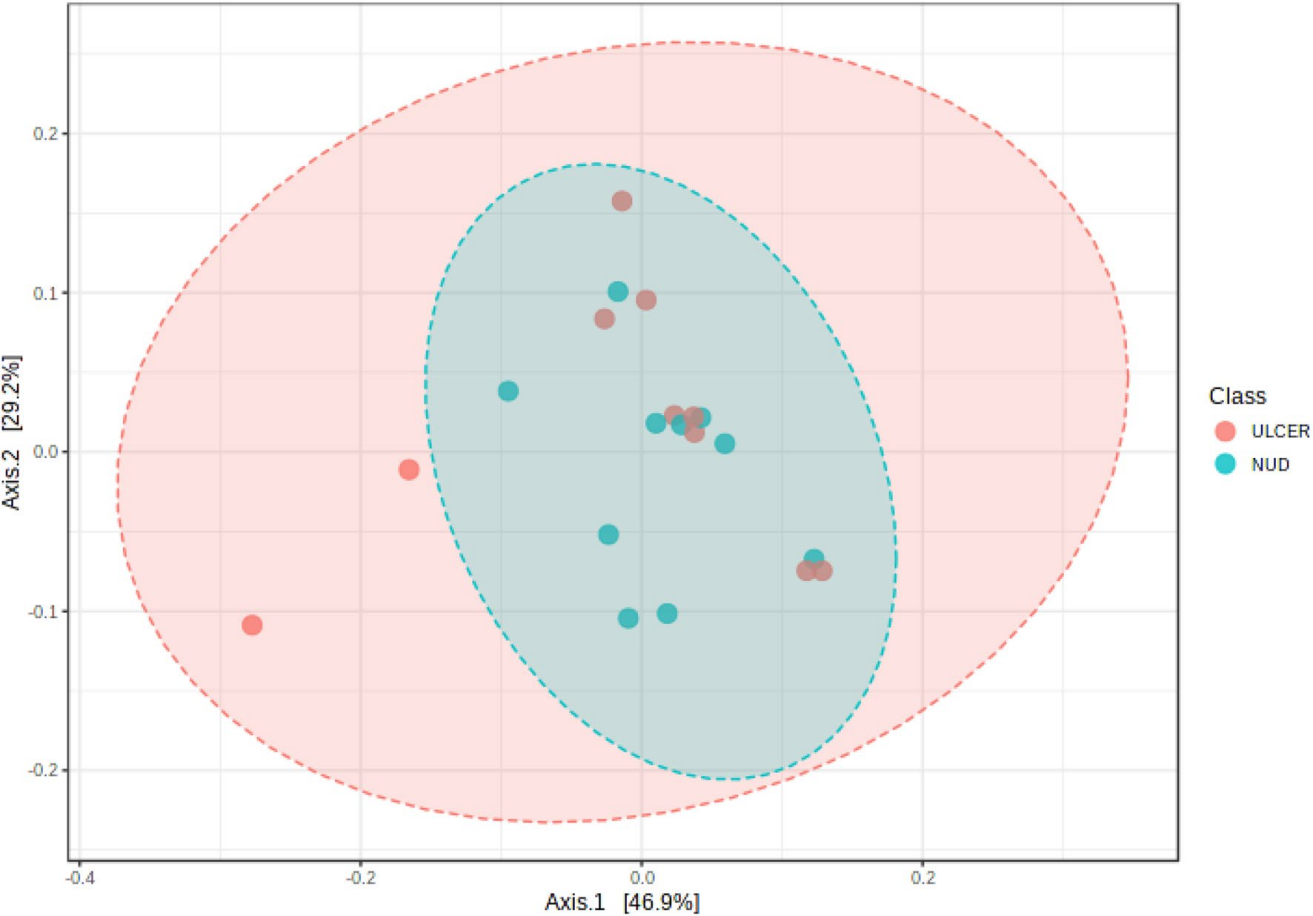
Beta diversity analysis of ulcer and the non-ulcer microbiome plotted in the Principal component analysis (PCoA) in 2D. Bray–Curtis distances were used to explain β-diversity between the two groups. (PERMANOVA) F value = 0.86; R^2^ = 0.045; p value < 0.51.

There was no significant difference in any specific bacterial abundance across the ulcer and non-ulcer groups. No difference was also observed in the alpha and beta diversity indices in both these groups (Figs. 6, 7). Bivariate Pearson correlation analysis showed a significant association between high redox potential, high gastric juice pH, and *H. pylori* abundance (Table 3). The tissue redox potential was negatively associated with *Pseudomonas caeni*, *Bacteroides acidifaciens*, and *Parabacteroides distasonis.* Organisms positively associated with GI bleeding include *Acinetobacter gulliouiae*, *Sulfuricurvum kujiense*, *Lactococcus garvieae*, *Butyriccoccus pullicaecorum*, and *Bifidobacterium longum*.

**Table 3:** Bivariate Pearson correlation analysis of species with respect to ORP, pH, and GI bleed.

Organism	Tissue ORP	Gastric juice pH	UGI bleed
*H. pylori*	0.491*	0.517*	− 0.304
*A. guillouiae*	− 0.206	− 0.117	0.510*
*S. kujiense*	− 0.209	− 0.121	0.458*
*P. caeni*	− 0.527*	− 0.486*	0.216
*L. garvieae*	− 0.260	− 0.138	0.464*
*B. pullicaecorum*	− 0.100	− 0.183	0.527*
*B. acidifaciens*	− 0.573**	− 0.098	0.068
*B. longum*	0.351	0.027	0.450*
*P. distasonis*	0.152	− 0.480*	− 0.606**

Significant values are in bold.

*p value < 0.01 **p value < 0.001.

## Discussion

Oxidative stress is implicated in the etiology of many acute and chronic diseases. Multiple oxidative stress biomarkers are available to indirectly infer the presence of reactive species, their end products, levels of enzymatic/non-enzymatic antioxidants, or the total antioxidant capacities. Tissue redox potential serves as a direct measure of oxidative stress as it accounts for both the antioxidants and prooxidants. It is preferably measured in vivo or immediately after sampling. The ORP influences the mucosa-associated microbiome. The anaerobes generally prefer a low ORP while the aerobes grow under high ORP conditions. This work is based on the premise that relative abundance microbial signatures may be used as a stable surrogate measure of ORP and thus oxidative stress. Such a global index precludes the need for measuring individual antioxidant and prooxidant chemical species. It also has an advantage over total antioxidant capacity measures, quantifying redox balance rather than antioxidant levels.

The role of *Helicobacter pylori* has been extensively investigated in the etiopathogenesis of peptic ulcer disease. Studies have shown that the redox state influences the pathogenic potential of *H. pylori*. Reducing agents like Bisulfite and glutathione have been shown to inhibit the growth of *H. pylori* and decrease gastric pathology^20,21^. Further, diet and other commensal flora influence the gastric redox state. The members of the *Prevotella* spp,* Propionibacterium* spp,* Faecalibacterium* spp,* Akkermansia* spp, etc., have also been documented in other studies. The gastric microbiota influences the progression of *H. pylori* gastritis^22^.

Thus, gastric mucosal health is a composite measure determined by tissue ORP rather than the presence or absence of a single organism *H. pylori*. The other commensal flora also have a bidirectional influence on the tissue ORP. There is a Spatio-temporal and inter-individual variability in the gut microbiome composition. Hence, it is hard to define a healthy gut mucosal microbiome^23,24^. The taxonomic composition of the microbiome is usually obtained through metagenomics. The functional significance of the consortium is derived from the taxonomic data. Although taxonomic diversity has been proposed as an indicator of a healthy microbiome, it is decoupled from functional diversity. It has been shown that the functional diversity of biomes can be best inferred from their profile of oxidoreductase genes which reflects the environmental ORP^25^.

The respiratory substrate used by an organism indicates the ORP of its niche. Atomic oxygen can exist in different redox states: molecular oxygen, superoxide, and hydrogen peroxide. Hydrogen peroxide is a reduced form of oxygen than superoxide and molecular oxygen. Molecular oxygen has the highest oxidizing state among the three. The enzymes using these forms of oxygen operate in different redox potential niches. In addition, the presence of low affinity or high-affinity cytochrome oxidase genes indicates the requirement of high or low levels of environmental oxygen/ORP, respectively^16^. The presence of enzymes that utilize oxygen, superoxide, and hydrogen peroxides as substrates are the basis for our hierarchial classification of organisms into redox groups. The microbiome structure is likely to be perturbed in pathology involving mucosal oxidative stress induced by the host or environmental factors. This perturbation can be readily appreciated when the organisms are categorized based on their redox properties.

Studies have shown that oxidative stress induces a predominant aerotolerant/facultative microbial community selection^26,27^. There is a decrease in obligate anaerobes in the presence of oxidative stress^28,29^. There is a positive correlation between the tissue ORP and the ratio of the most abundant aerobic group-I organisms over all the anaerobic groups (III, IV, and V-b) except the V-a group (Fig. 1). The V-a group consisting of Clostridial species and the genera are inversely correlated with the other anaerobic groups (Figs. 4, 5). Direct measurement of oxidative stress by measuring ORP and its correlation with the Microbial Redox Index (aerobe/anaerobe ratio) is the salient feature of our study. A similar but composite Metagenomic Aerotolerance Predominance Index (MAPI) has been proposed in the context of the fecal specimen from the lower gut in malnourished children^30^. The MAPI index is based on the ratio of the relative abundance of aerotolerant species over obligate anaerobes.

Shannon diversity and species richness measures are considered surrogate markers of a healthy microbiome. However, in gastric mucosa, *H. pylori*-induced atrophic gastritis has been documented to harbor more diverse organisms than non-atrophic gastritis^31^. Further, Intestinal metaplasia is associated with decreased diversity compared to chronic gastritis. Since our study population consisted of a small number of patients, observation related to subsets of histopathological entities could not be verified. Nevertheless, an inverse relationship between the Shannon index/species richness and the Microbial Redox Index suggests decreased diversity with increased oxidative stress (Figs. 2, 3).

Bivariate Pearson correlation analysis showed the expected association between *H. pylori* and tissue ORP (Table 3). Similarly, the negative association between ORP and the anaerobic organisms *Bacteroides acidifaciens* and *Parabacteroides distasonis* may be explained. Further studies are required to address the relationship between Pseudomonas caeni and ORP. The association between GI bleed and the presence of certain species also needs to be validated in more extensive studies. The sample size limitation also precluded comparison across ulcer and non-ulcer microbiomes (Figs. 6, 7). Our study is based on 16s rRNA profiling of the gastric tissue bacteria in chronic gastritis. A metagenomic approach could have yielded additional data to validate other observations. Further studies are required to overcome the sample size limitations and non-availability of healthy control groups.

## Conclusion

This study describes an elegant method to quantify mucosal oxidative stress and thereby mucosal health based on prior knowledge of a few predominant bacterial species/genera and their placement in the redox spectrum. The approach described helps diagnose oxidative stress in pathological states and is widely applicable to other body sites. The positive and negative correlations between organisms within and across redox groups will unravel hitherto undiscovered functional networks. It can also be a valuable tool in evaluating prebiotic, probiotic, and other therapeutic interventions^32^ targeted at reducing the inflamed gastric mucosa's redox state (Eh). The therapeutic effect of electrochemically reduced water can be evaluated using Microbial redox mapping and Microbial redox index described herein. Redox community-based microbiome analysis may also help personalize antibiotic, prebiotic and probiotic interventions.

## Data Availability

All sequencing data are publicly available on the sequence Read Archive (SRA) under the study accession number PRJNA684564.
